# Spatio-temporal patterns of human-wildlife conflicts and effectiveness of mitigation in Shuklaphanta National Park, Nepal

**DOI:** 10.1371/journal.pone.0282654

**Published:** 2023-04-17

**Authors:** Bindu Pant, Hari Prasad Sharma, Bhagawan Raj Dahal, Sandeep Regmi, Jerrold L. Belant

**Affiliations:** 1 Central Department of Zoology, Institute of Science and Technology, Tribhuvan University, Kirtipur, Kathmandu, Nepal; 2 Zoological Society of London Nepal Office, Kathmandu, Nepal; 3 Department of Fisheries and Wildlife, Michigan State University, East Lansing, Michigan, United States of America; Universitat Autònoma de Barcelona, SPAIN

## Abstract

Human-wildlife interactions occur where human and wildlife coexist and share common resources including food or shelter. Increasing wildlife populations within protected areas also can increase interactions with humans living adjacent to these areas, resulting in conflicts including human casualty, livestock depredation, crop damage, and property loss. We analyzed six years human-wildlife conflict data from 2016–2021 in the buffer zone of Shuklaphanta National Park and conducted questionnaire survey to investigate factors influencing human-wildlife conflicts. Nineteen people were attacked by wildlife, primarily wild boar (*Sus scrofa*). Ninety-two livestock were killed by leopard (*Panthera pardus*), and among these most were sheep or goats killed near ShNP during summer. Crops were most frequently damaged by Asian elephants (*Elephas maximus*), followed by wild boar. Greatest economic losses were from damage to rice, followed by sugarcane and wheat. Asian elephant was the only reported species to cause structural damage to property (e.g., homes). Majority of respondents (83%) considered that the mitigation techniques that are currently in practice are effective to reduce the conflicts. However, the effectiveness of the mitigation techniques are the species specific, we recommend use of more efficacious deterrents (e.g., electric fencing) for large herbivores and mesh wire fencing with partially buried in the ground. Effective collaboration among different tiers of government, non-governmental organizations, civil societies and affected communities are important to share the best practices and continue to apply innovative methods for impactful mitigation of human-wildlife conflicts in the region.

## Introduction

Human-wildlife interactions occur when human and wildlife live in close proximity [1] and share common resources including food and shelter [2]. Rapid human population growth and competition for resources has increased the frequency of conflicts with wildlife [3], exacerbated in and near protected areas where wildlife is often more abundant [4]. Conflicts were mainly due to increasing wildlife crime associated with increased demand for wildlife and their body parts [5], livestock and crop depredation, attacks on humans, and property damage [6]. Losses from wildlife including crop damage [7], livestock depredation [8], and human injuries or death [9, 10] increase negative attitudes of people toward wildlife [11]. These negative attitudes can adversely affect many wildlife species due to retaliatory killing [11, 12].

Human-wildlife conflicts are generally more severe to farmers who live in rural communities [8, 13, 14]. These individuals can lose 10–15% of their total agricultural production from wildlife damage [15, 16]. For example, in Bardia National Park, Nepal, 70% of respondents interviewed experienced crop damage by Asian elephants (*Elephas maximus*) [17]. In Chitwan National Park, Nepal, crop damage represented 70% of all human-wildlife conflicts reported during 2013–2017 [18].

However, human injury or death from wildlife attacks is also a serious form of conflict worldwide. For example, more than 100 people are killed annually by Nile crocodiles (*Crocodylus niloticus*) in Mozambique and Namibia [15] and in Tanzania during 1990–2004 more than 563 people were killed and about 308 injured from lions (*Panthera leo*; [19]). In Nepal, attacks on people are largely by large mammals including tigers (*P*. *tigris*; [20]), leopards (*P*. *pardus;* [21, 22]), greater one-horned rhino (*Rhinoceros unicornis;* [23]), Asian elephants; [24]) and Asiatic black bears (*Ursus thibetanus*; [25]). Around 1139 large mammal and 887 human deaths were recorded in Nepal between 2000–2020 [10] which demonstrates the serious threats on wildlife survival and human mortality. In addition, almost 800 incidents of crop damage by Asian elephants (n = 517) and greater one-horned rhino (n = 280) and 357 livestock depredation events by tigers (n = 77) and leopards (n = 280) were reported from Nepal annually [10]. In protected areas of Nepal, the incidences of wildlife attacks can also threaten human livelihood, for example, 4,014 wildlife incidents with humans, livestock, crops, and other property damage occurred in Chitwan National Park [26]. In contrast, in Shuklaphanta National Park, only one human death and five injuries were recorded during 2016–2020 [27, 28]. These conflicts typically occur when wildlife enter human settlements due to human expansion into areas of suitable habitat [29].

Natural areas are also increasingly exploited due to increasing human populations which has altered the global landscape [30]. The rise in human population in Nepal (e.g., 14% during 2001–2011 [31]) has resulted in encroachment of wildlife habitats, the compression of species into small habitat areas, and direct competition with local communities [32]. Illegal and unmanaged settlements in and around protected area can hinder conservation and activities such as logging, grazing, and poaching have harmed wildlife populations and their habitat [32].

Various management tools such as deterrents, barriers and lethal control are used to mitigate human-wildlife conflicts globally [33]. Numerous methods are also used in Nepal to reduce the frequency and severity of human-wildlife conflicts. For example, electric fencing can be highly effective in reducing crop damage [34]. Other exclusion and deterrent techniques include constructing livestock pens with barbed-wire [35] and cultivating unpalatable crops [36]. Compensatory and incentive-based programs also have been used in local communities to reduce costs of human-wildlife conflicts such as community-based conservation and integrated conservation and development projects [34, 35, 37, 38]. Nepal Government paid >400,000 US dollar to the victim between 1998 and 2016 as a relief in Chitwan National Park, Nepal [26]. Despite some differences between these compensatory and incentive-based programs, the term incentive-based programs have been used to describe projects intended to balance conservation with the livelihoods of local residents near protected areas [35, 39].

With recent incidents including elephant attacks resulting in multiple human deaths and injuries [29] and a human fatality from a tiger attack [40], human-wildlife conflicts are becoming an increasingly serious issue in and near Shuklaphanta National Park (ShNP), Nepal. Since ShNP encompasses a large area including nine community-use buffer zones and 42 community forests, it is critical to local people’s livelihoods. Human-wildlife conflicts in ShNP have reportedly resulted in negatives attitude toward wildlife by local people [29], but the frequency and magnitude of conflicts and mitigation techniques used have not been characterized adequately. Our objective was to quantify the type, frequency, and timing of human-wildlife conflicts and to identify measures taken by ShNP personnel to address these conflicts.

### Study area

**Fig 1:**
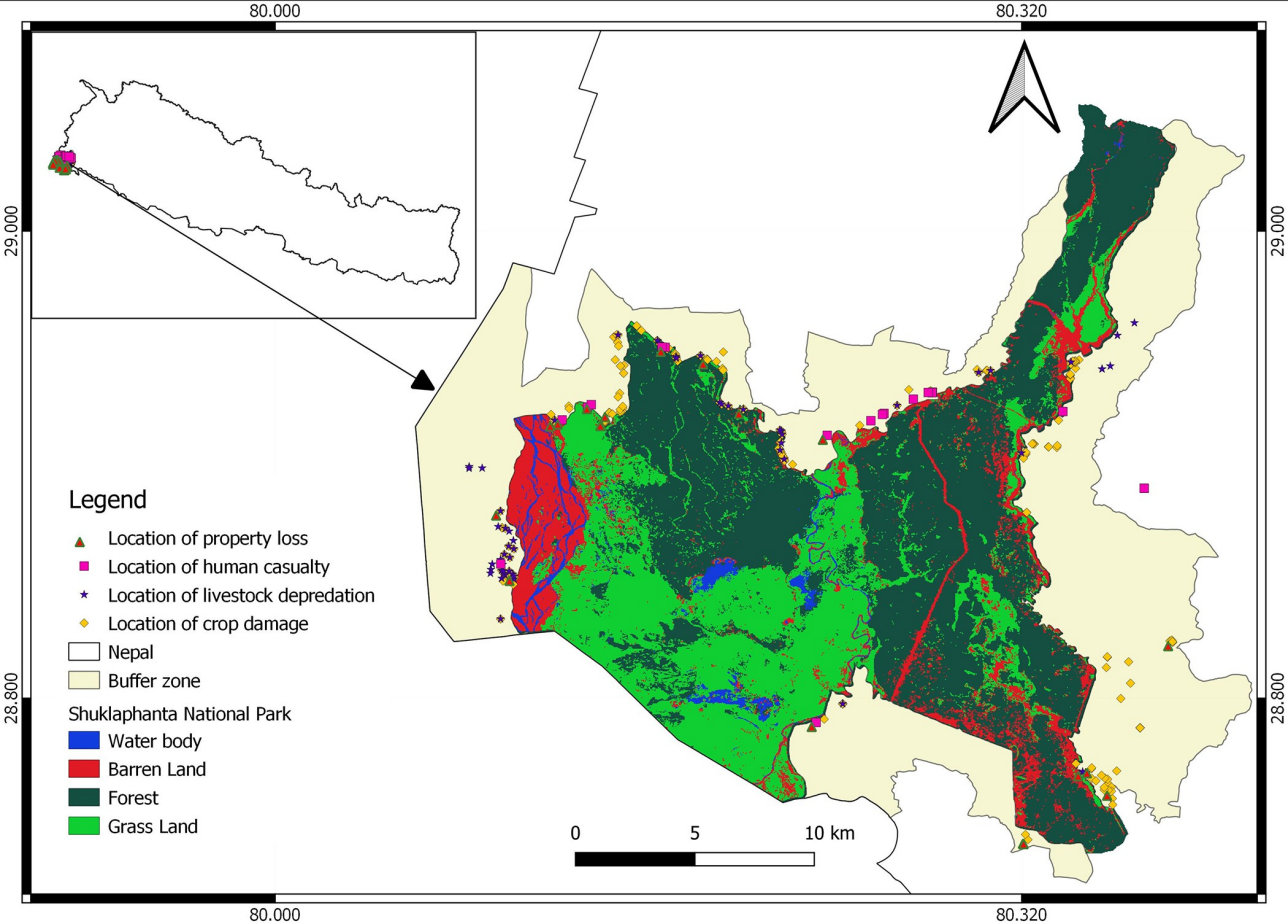
Shuklaphanta National Park, Nepal, with adjacent buffer zones and locations of human-wildlife conflicts, 2016–2022.

Shuklaphanta National Park (28°45’47"-29°02’52"N and 80°05’45"-80°21’43"E) comprises 305 km^2^ in southwestern Nepal (Fig 1). The park was established as a Wildlife Reserve in 1979 before being designated a national park in 2017. The park is bounded by settlement forests and the Dadeldhura District in the north, the Syali River in the East, the Indian border (Grasslands of Pilibhit Tiger Reserve and Lagga Bagga forests) in the south, and the Mahakali River in the west. Most of ShNP occurs in the Gangetic floodplain with elevations from 175 to 1300 m above sea level. The climate is tropical monsoon with average monthly minimum and maximum temperatures of 7 and 37 C, respectively. The park area has more than 665 plant species [41], 56 reptiles, 15 amphibians, 423 bird and 53 mammal species [42]. Mammal species include sloth bear (*Melursus ursinus*), leopard, Asian elephant, wild boar (*Sus scrofa*), and greater one-horned rhino [41].

The buffer zone of ShNP contains 34 wards within five Municipalities and two Rural Municipalities including Bhimdatta Municipality (Ward No. 13−19), Bedkot Municipality (Ward No. 9 and 10), Belauri Municipality (Ward No. 9), Mahakali Municipality (Ward No. 1−10), Shuklaphanta Municipality (Ward No. 1−7 and 12), Beldadi Rural Municipality (Ward No. 2,4 and 5) and Laljhadi Rural Municipality (Ward No. 3, 4 and 6). There are about 27067 households in the study area [43] and major occupations are laborer and agriculture. Important crops grown include wheat, corn, sugarcane, and bananas; dominant livestock includes cattle, buffaloes, goats, sheep, and pigs.

## Methods

We collected secondary and primary data on wildlife attacks. Secondary data included household size, the number of wildlife attacks on humans, livestock, crops, and property and economic losses from 2016–2021, available from ShNP office. We collected names and locations of 900 households who reported their losses or casualties to ShNP officials. For primary data, we conducted a questionnaire survey with affected local people. Prior to fieldwork and interview, we also received written consents from the Department of National Parks and Wildlife Conservation (Permission letter number 1619) and written consents from participating individuals.

From these 900 household we selected 300 households for the questionnaire survey by random sampling method using the rand() command in MS excel [44]. Though the minimum required sample size is 270 households for a 95% confidence interval with 5% margin of error, we selected 300 households at random across 34 wards (Bhimdatta Municipality Ward No. 13−19, Bedkot Municipality Ward No. 9 and 10, Belauri Municipality Ward No. 9, Mahakali Municipality Ward No. 1−10, Shuklaphanta Municipality Ward No. 1−7 and 12, Beldadi Rural Municipality Ward No. 2,4 and 5 and Laljhadi Rural Municipality Ward No. 3, 4 and 6 for the survey. We conducted the survey during 28 September 2021–8 January 2022, interviewing one person >18 years old from each household; individuals selected were not based on education level, gender, ethnicity, or religion. We requested demographic data including age, gender, education level, family size, religion, and socio-economic conditions. Whenever possible, we interviewed people who witnessed the conflict or were victims of attacks on humans and requested they show us the site where the incidents occurred, then recorded the location using hand-held GPS. In the event of wildlife attacks resulting in death, we interviewed the victim’s relative or an eyewitness.

We asked whether respondents experienced conflicts from wildlife including attacks on humans, livestock or crop depredation, or property loss. We also asked whether mitigation measures were implemented following conflicts and if these measures reduced conflicts. We categorized conflicts as attacks on humans (death or injury), livestock depredation (buffalo, cattle/oxen, goat/sheep), crop depredation (wheat, rice, corn, mustard, potato, or sugarcane), or property loss. We used Chi-square and Man-Whitney U Tests for categorical and continuous data, respectively.

We used generalized linear models to identify factors affecting livestock and crop depredation. For livestock depredations, we used the total reported number of livestock killed during 2016–2021 as the response variable and predictive variables were number of livestock owned (cattle/ox, buffalo, and goat/sheep), distance (adjacent [<500 m] or distant [≥500 m]) to ShNP, season (summer [May–October] or winter [November–April]) and whether mitigation measures (e.g., electric fencing, wire, and barbed wire) were conducted by ShNP personnel (yes or no). We categorized distance from the park boundary as near (500 m) and far (>500 m) because the livelihood of the people living near and far from the park is influenced by park activities. For crop damage, we used total crop damage for the response variable, and predictive variables including hectares of land owned, distance to ShNP, whether mitigation measures were conducted by ShNP personnel, and whether preventive measures (e.g., setting fires, and noise making including use of fire crackers) were conducted by the respondents (yes or no). We compared models using Akaike’s information criterion for small samples (AICc) and model weights [45] and selected the model for livestock and crop depredation with lowest AICc score. We estimated economic loss using the market value of the loss from the compensation log book of ShNP. We estimated the monetary value of crop loss based on the annual sale price of each crop, then converted livestock and crop losses to USD using the Nepal Rastra Bank’s exchange rate. We performed all analyses in program R [46] with significance established using α = 0.05.

## Results

Most respondents (77.6%, n = 233) lived near ShNP. There were no differences in attributes of respondents based on gender, education, age, or occupation living adjacent or distant to the park boundary (Table 1). However, people adjacent to ShNP owned more livestock (median = 5) than people living distant (median = 4) (Man-Whitney Test, W = 9129.5, p = 0.033). Overall, we documented 626 reported human-wildlife conflicts with 78% adjacent to ShNP and conflicts occurring up to 5 km from the park boundary.

**Table 1:** Demographic and social information of respondents (n = 300) living adjacent (<500 m) and distant (≥ 500 m) to Shuklaphanta National Park, Nepal. Parameters include age (years), gender (male or female), education (educated represents people who attended school through grade five or above; uneducated did not attend school), agriculture-based livelihood (people whose primary vocation is agriculture), family size (number of people), and livestock owned (number of all hoofed livestock owned). Values in parentheses are ranges.

Parameter	Adjacent	Distant	Statistics
Median age	40 (18–85)	40 (20–90)	Mann Whitney test, W = 7472, p = 0.594
Gender (male %)	51	49	χ^2^ = 0.069, df = 1, p = 0.793
Educated (%)	42	44	χ^2^ = 0.157, df = 1, p = 0.692
Agriculture-based livelihood (%)	93	96	χ^2^ = 0.501, df = 1, p = 0.479
Median family size	6 (1–34)	6 (2–23)	Mann Whitney test, W = 8879.5, p = 0.084
Median number of livestock owned	4 (0–20)	5 (0–25)	Mann Whitney test, W = 9129.5, p = 0.033

### Human casualties

Wildlife attacked 19 people including 9 injuries and 1 death from wild boars adjacent to ShNP, 5 (4 adjacent) by Asian elephants, 3 by leopards distant to ShNP, and 1 near ShNP by a greater one-horned rhino. Ten victims were male and 9 were female; average victim age was 46 years old (range = 11–76 years). More attacks occurred during summer (14 adjacent) than in winter (1 adjacent, 4 distant) (χ^2^ = 14.632, df = 2, p < 0.001). Attacks were near homes or agricultural land (7 each), 3 were along roads, and two were within ShNP. More attacks on humans occurred during the day (11 adjacent, 4 distant) than at night (4 adjacent).

### Livestock depredation

**Fig 2:**
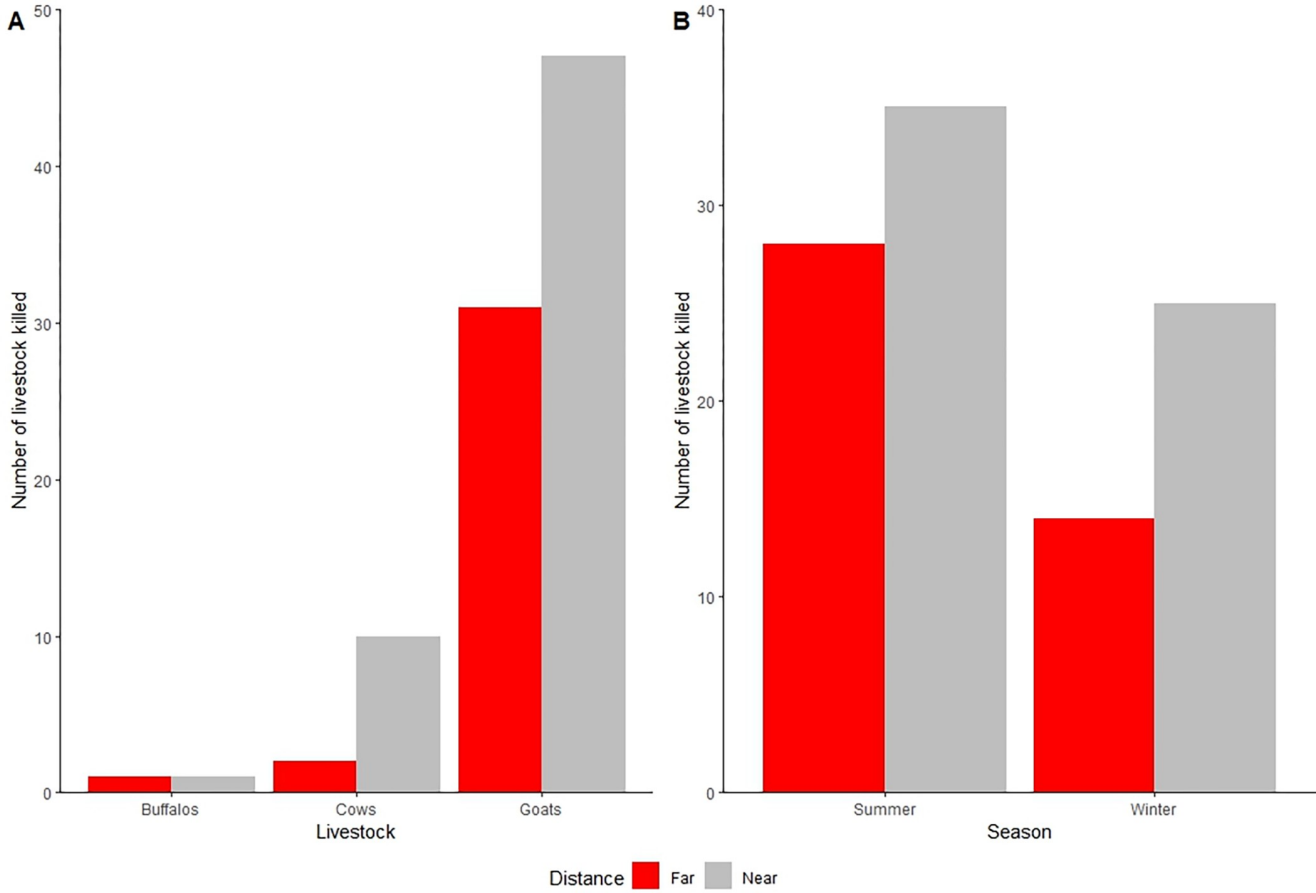
Number of livestock depredation (A); and seasonal livestock depredation (B) near (<500 m) and far (>500 m) from the Shuklaphanta National Park boundary between 2016 and 2022.

Leopard was the only species involved in livestock depredations, with most attacks (64.0%, n = 58) adjacent to ShNP. Of 92 livestock depredations, 84.8% were goats/sheep, followed by cattle, and buffalo (Fig 2). More reported depredations occurred in summer (57.6%) than in winter (42.4%). The livestock depredation was increased with increasing the number of livestock in summer season at low practices area of mitigation measures (Table 2).

**Table 2:** Model-averaged parameter estimates with lower (LCL) and upper (UCL) 95% confidence limits describing livestock depredations by wildlife in Shuklaphanta National Park, Nepal, 2016–2021. Number of livestock killed was used as the response variable and total number of livestock owned, season (summer and winter), distance from the park (near: < 500 m and far: >500 m from the park boundary) were predictor variables. From which best supported model and successive models were generated and parameter estimates were averaged from all models. Significant effects are in bold.

Parameter	Estimate	LCL	UCL	SE	P
(Intercept)	-2.465	-3.149	-1.834	0.335	**<0.001**
Total livestock owned	0.076	0.022	0.125	0.026	**0.004**
Season	1.629	1.366	1.911	0.138	**<0.001**
Distance from park	-0.391	-0.816	0.050	0.220	0.076
Mitigation measures	-0.318	-0.800	0.193	0.252	0.207

### Crop damage

Most respondents cultivated rice (n = 264), followed by wheat (n = 263), potato (n = 238), mustard (n = 235), maize (n = 223) and sugarcane (n = 51). Of the 267 respondents that grew crops, 96.4% (209 adjacent, 48 distant) experienced crop damage. More reported crop depredations occurred at night (n = 233) than during day (n = 48); crop damage occurred from germination to harvest, with most damage during harvest.

**Fig 3:**
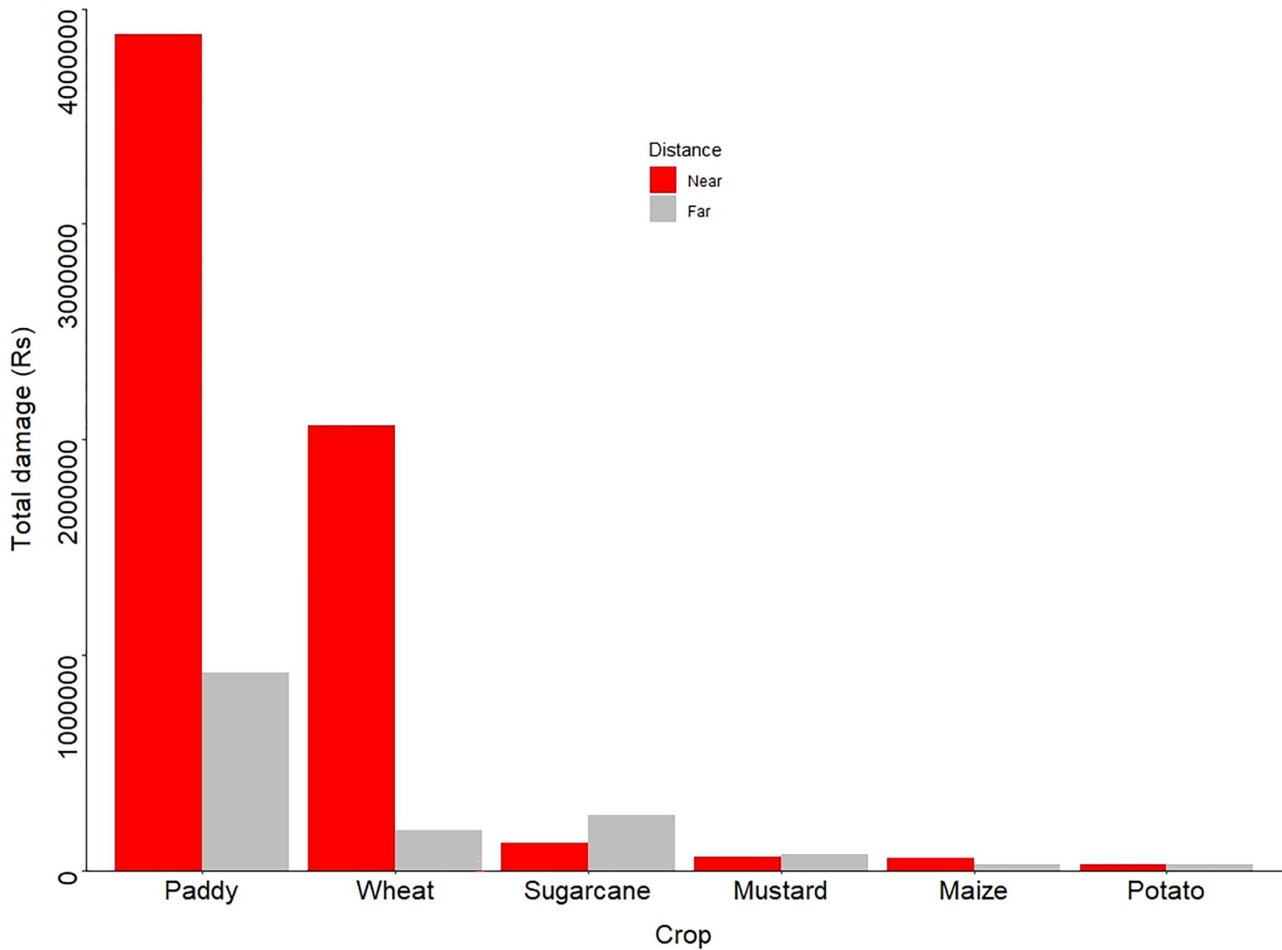
Total amount of crop damage near and far from the park Shuklaphanta National Park between 2016 and 2022.

The number of crop raiding events decreased distant from ShNP. However, the likelihood of crop damage increased with increasing number of mitigation measures, area under production, but did not with increased use of preventative measures (Table 3). Greatest overall economic losses of crops were by Asian elephants, followed by wild boars (Table 4, Fig 3). Overall economic loss to farmers was estimated to be USD 103,335 including rice (USD 41,608), followed by sugarcane was USD 39,990, wheat (USD 19,412), mustard (USD 1,138), corn (USD 701) and potato (USD 484) during 2016–2022.

**Table 3:** Model-averaged parameter estimates with lower (LCL) and upper (UCL) 95% confidence limits describing crop depredations by wildlife in Shuklaphanta National Park, Nepal, 2016–2021. Significant effects are in bold font.

	Estimate	SE	LCL	UCL	Z	P
Intercept	6.836	0.018	6.801	6.826	375.46	**<0.001**
Land owned	0.009	<0.001	0.081	0.098	20.13	**<0.001**
Distance near	0.089	0.004	0.009	0.100	164.90	**<0.001**
Mitigation measures	0.017	0.004	0.009	0.025	4.30	**<0.001**
Preventive measures	-0.159	0.018	-0.193	-0.124	8.87	**<0.001**

**Table 4:** Estimated value of reported crop damage (in USD) by wildlife, Shuklaphanta National Park, Nepal, 2016–2021.

Species	Wheat	Rice	Corn	Mustard	Potato	Sugarcane
Asian elephant	8,639	28,597	375	278	−	39,954
Wild boar	9,324	11,096	326	860	484	−
Nilgai	813	527	−	−	−	36
Greater one-horned rhino	−	948	−	−	−	−
Spotted deer	458	361	−	−	−	−
Rhesus monkey	180	79	−	−	−	−
Combined	19,413	41,608	701	1,138	484	39,990

### Property damage

Asian elephants reportedly damaged property on 22 occasions, including 19 homes, 2 sheds, and 1 oil seed store, with overall estimated economic loss of USD 5972. All incidents occurred at night and damage was more severe adjacent to ShNP. No other species were reportedly involved with property damage.

### Effectiveness of mitigation measures

**Fig 4:**
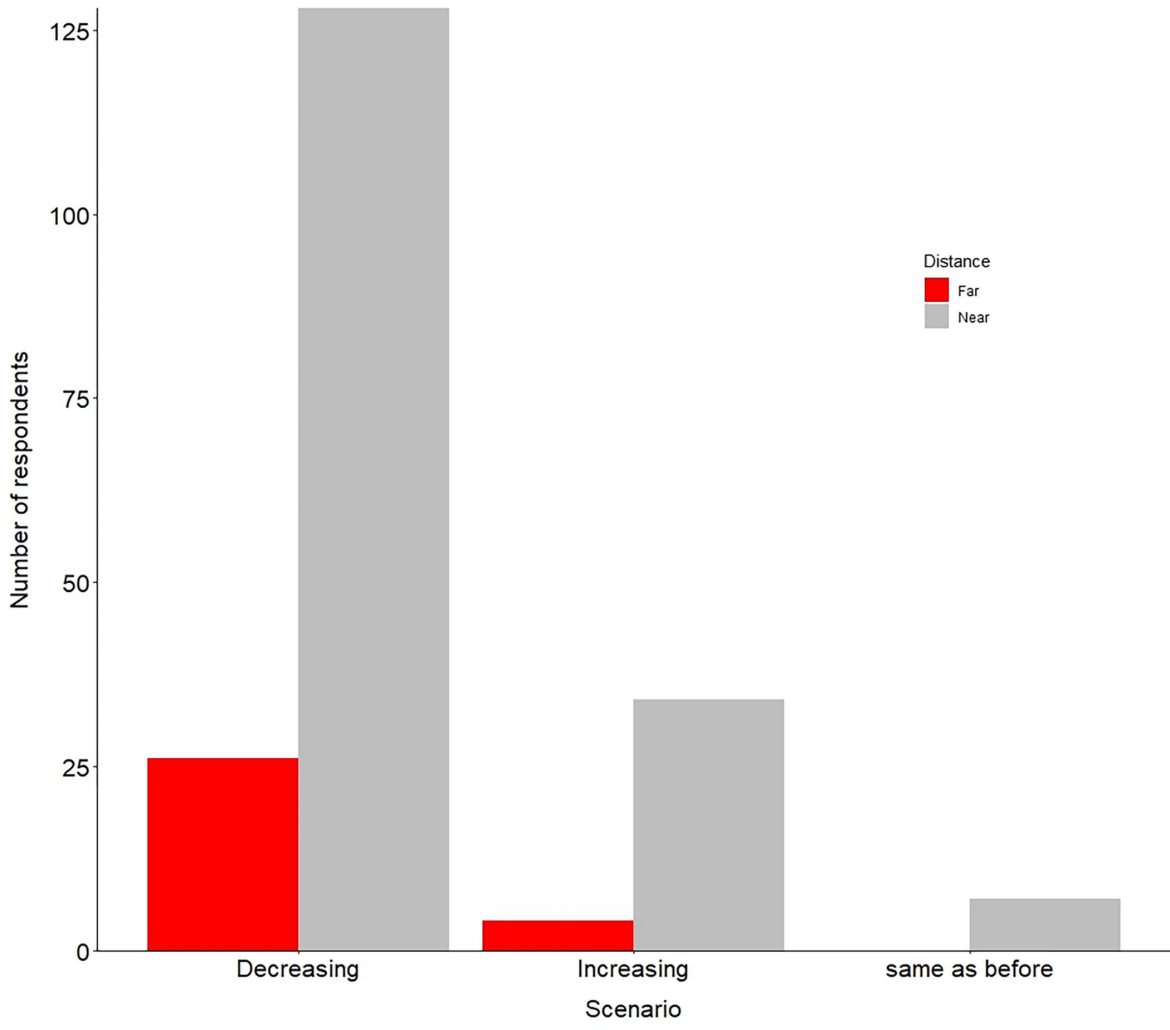
Number of respondent’s perception towards the scenario of human-wildlife conflict after applying mitigation measures near and far from the Shuklaphanta National Park between 2016 and 2022.

Of the 300 respondents, 65.7% reported that conflicts were reduced after implementinge mitigation measures, while 28.0% said it increased increasing, and 6.3% respondents said no change (Fig 4). Two hundred and four respondents had knowledge of mitigation measures used by the park. More respondents 76.9% (n = 154, near = 128, far = 26) agreed there were decreased conflicts with wildlife due to the effective implementation of mitigation measures. However, 18.4% (n = 38, near = 34, far = 4) respondents mentioned increased conflicts, and 1.7% (n = 7, near = 7) reported no change (Fig 4).

## Discussion

The magnitude of human-wildlife conflict was greater in the proximity to the National Park. Most people living here depend on agriculture for their livelihoods, which in turn relies on adjacent natural resources and increases human-wildlife conflicts [47]. That males and females are directly involved in agricultural activities, including equal involvement in protecting crops from damage, supports the similar numbers attacked by wildlife. Wildlife attacks generally occurred during the day when people are outside working on their farms, similar to previous studies of wildlife attacks on humans [48, 49].

Most conflicts were carnivore attacks on human and livestock by carnivores, which results in negative attitudes toward them [50]. In addition to carnivores, the wild boar was the species most frequently involved in attacks on humans, potentially due to their greater abundance in protected areas [51–53] across the country. Most attacks occurred during summer in agricultural lands and near homes might be due to few grazing areas inside the park [54], or that wild boars move to open habitat near human settlements during summer to forage on crops [55]. That attacks were more frequently distant from ShNP is likely due to wild boars seeking forage in buffer zone forests and thus increasing the chances of interactions with humans [56, 57]. Further, respondents described wild boars as acting aggressively when people attempted to deter them from cultivated areas.

Most livestock depredations occurred adjacent to ShNP; populations of carnivores are increasing across protected areas throughout Nepal [58]. Greater frequency of livestock depredations during summer is likely related to the increased time livestock spend grazing in pastures [59], compared to being kept in enclosed sheds during winter. Goats were most frequently depredated by leopards in part because of their greater abundance. Further, leopards preferred prey range in size from 2 to 40 kg [60, 61] and across domestic prey species, leopards select smaller-sized hoofed animals such as goats and sheep [62, 63]. Livestock depredation by wildlife also occurs in areas where livestock can free-range or when shelters are poorly constructed and facilitate access by carnivores like leopards [64]s. Improved animal husbandry practices including secure enclosures, installing stronger fencing, and providing stall feeding could reduce incidents of wildlife predation on livestock [65].

Abundance of wildlife increased following establishment of ShNP [27], while the amount of forest cover during this period has declined [24]. This dynamic of increasing wildlife and decreasing forest area has resulted in increased foraging use of cultivated lands by wild herbivores, likely due in part to reduced natural forage [66]. Before the 1950s, most of the Tarai Region of Nepal was unoccupied due to the high risk of malaria. However, after eradication of malaria and government resettlement schemes, the human population in this region has increased exponentially [67]. That crop damage was more common adjacent to ShNP might be due to the higher forest cover in the edge of ShNP [68]. Encroachment into elephant habitat by humans is a leading cause of crop damage by Asian elephants [69]. Asian elephants in this study likely caused property damage and injured people while searching for food [70].

Wild boars frequently damaged agricultural crops, likely because of their high abundance in and around the protected areas of Nepal [51] and preference for agricultural crops over native forage [71]. The most frequently damaged crop in our study area was rice, which is selected over other crops and grown extensively throughout Nepal [28, 72]. Further, previous studies in human wildlife conflicts in ShNP found that damage to rice accounted for nearly 70% of total crop loss [73]. Most crops were damaged by wildlife during harvest when their nutritional value [74, 75] and palatability [72] are greater.

In contrast to our expectations, the likelihood of crop damage increased with the increased number of mitigation measures. This might be due to incomplete or inconsistent use of mitigation measures or a consequence of areas otherwise experiencing frequent conflicts and higher amounts of loss, or that mitigation measures are resolving human-wildlife conflict locally but shifting the problems to adjacent areas [76]. Similarly, though mitigation measures may reduce the impact of large mammals like elephant and rhino, they are appear less effective for wild boar, deer, and other smaller-bodies mammals [34].

Based on the results of this study we offer several recommendations to mitigate wildlife damage. To reduce wildlife attacks on humans, timely identification and management of wildlife and public awareness program should be implemented [38]. Further, establishment of well-equipped and prepared emergency rescue teams, awareness by local people of conflict prone areas and areas of species movements, and enforced regulation and potential limitations of anthropogenic activities in and around protected areas should be considered [32]. To reduce livestock losses, we recommend improved animal husbandry practices such as night-penning and consideration of livestock insurance from a government managed relief fund which could include compensation limits [35–37]. Adopting alternative crops that are less palatable or bee keeping could reduce crop damage by some species [36]. Electric fencing could also be used effectively to reduce all forms of conflicts identified in this study [34]. These and other techniques implemented in an integrated approach are likely needed to maximize reduction of human-wildlife conflicts in and near ShNP.
